# Spatial variability of organic matter properties determines methane fluxes in a tropical forested peatland

**DOI:** 10.1007/s10533-018-0531-1

**Published:** 2018-11-26

**Authors:** N. T. Girkin, C. H. Vane, H. V. Cooper, V. Moss-Hayes, J. Craigon, B. L. Turner, N. Ostle, S. Sjögersten

**Affiliations:** 1 School of Biosciences, University of Nottingham, Nottingham, NG7 2RD UK; 2 British Geological Survey, Centre for Environmental Geochemistry, Keyworth, NG12 5GG UK; 3 Smithsonian Tropical Research Institute, Apartado, 0843-03092 Balboa, Ancon Republic of Panama; 4 Lancaster Environment Centre, Lancaster University, Lancaster, LA1 4YQ UK

**Keywords:** Tropical peat, Carbon dioxide, Methane, Organic matter, Rock-Eval pyrolysis, Geochemistry

## Abstract

Tropical peatland ecosystems are a significant component of the global carbon cycle and feature a range of distinct vegetation types, but the extent of links between contrasting plant species, peat biogeochemistry and greenhouse gas fluxes remains unclear. Here we assessed how vegetation affects small scale variation of tropical peatland carbon dynamics by quantifying in situ greenhouse gas emissions over 1 month using the closed chamber technique, and peat organic matter properties using Rock-Eval 6 pyrolysis within the rooting zones of canopy palms and broadleaved evergreen trees. Mean methane fluxes ranged from 0.56 to 1.2 mg m^−2^ h^−1^ and were significantly greater closer to plant stems. In addition, pH, ranging from 3.95 to 4.16, was significantly greater closer to stems. A three pool model of organic matter thermal stability (labile, intermediate and passive pools) indicated a large labile pool in surface peat (35–42%), with equivalent carbon stocks of 2236–3065 g m^−2^. Methane fluxes were driven by overall substrate availability rather than any specific carbon pool. No peat properties correlated with carbon dioxide fluxes, suggesting a significant role for root respiration, aerobic decomposition and/or methane oxidation. These results demonstrate how vegetation type and inputs, and peat organic matter properties are important determinants of small scale spatial variation of methane fluxes in tropical peatlands that are affected by climate and land use change.

**Electronic supplementary material:**

The online version of this article (10.1007/s10533-018-0531-1) contains supplementary material, which is available to authorized users.

## Introduction

Plant inputs of leaf and stem material and rhizodeposits are important in regulating greenhouse gas (GHG) emissions from tropical peat (Hoyos-Santillan et al. 2016b). Land use and climate change can significantly alter plant community composition, which may alter peat chemistry through changes in species-specific carbon inputs (Tonks et al. 2017; Girkin et al. 2018a, b). The consequences of this change are likely to be pronounced as peatlands contain an estimated 15–19% of the global peat carbon stock, and are a significant source of carbon dioxide (CO_2_) and methane (CH_4_) (Page et al. 2011; Dargie et al. 2017).

Tropical peats derived from different botanical origins have distinct organic matter chemistries which results in different decomposition rates and GHG production (Hoyos-Santillan et al. 2015), particularly in comparison to temperate and boreal peatlands where peat formation is predominantly driven by graminoid and moss inputs (Turetsky et al. 2014). Under water-logged anoxic conditions that typify peatlands, decomposition is associated with significant CH_4_ fluxes. CO_2_ is also produced through a combination of autotrophic root respiration, aerobic heterotrophic microbial respiration and the oxidation of CH_4_ (Sayer and Tanner 2010). Differences in vegetation can therefore affect the balance of these emissions through changes in substrate inputs, organic matter composition, microbial communities (Ayres et al. 2009; Keiser et al. 2014), root release of oxygen (Hoyos-Santillan et al. 2016a), and physical properties of the peat (Wright et al. 2013b), and through providing a significant pathway for GHG transport (Pangala et al. 2017).

The extent of plant influences can vary spatially, with rhizosphere soils subject to strong root influences featuring distinct physiochemical properties compared to bulk soils. For example, phosphorus (Hinsinger and Gilkes 1996) and nitrogen limitation (Landi et al. 2006) are known to drive the release of root exudates, which may in turn be associated with changes in soil properties such as pH (Youssef and Chino 1989), as well as changes in microbial activity and composition (Gomes et al. 2003; Shi et al. 2011). Combined with differences in biogeochemical properties and microtopography between plant species, peats can display significant small scale (cm to m) variation in chemical properties (Jauhiainen et al. 2005; Wright et al. 2013b). However, the extent to which this variation in peat chemistry under individual plants influences carbon cycling in tropical peatlands is unclear.

Rock-Eval pyrolysis has previously been used to characterise carbon cycling and organic matter transformation within mangrove (Marchand et al. 2008) and marine sediments (Hare et al. 2014), as well as soil organic matter (SOM) dynamics with profile depth (Disnar et al. 2003; Sebag et al. 2006, 2016), and the extent of decomposition and humification in northern peats (Delarue et al. 2013; Biester et al. 2014). Parameters from Rock-Eval of particular relevance to peat organic matter include the hydrogen index (HI), indicative of atomic H:C, and the oxygen index (OI), indicative of atomic O:C, in addition to TpkS2, the temperature associated with the highest yield of bound hydrocarbons, and S2, a measure of overall hydrocarbons released on the thermal cracking of organic matter for temperatures up to 650 °C. Higher values of HI in peat deposits have been interpreted as indicating the presence of hydrogen-rich labile carbon compounds predominantly found in fresh organic matter inputs (Outridge and Sanei 2010). HI has previously been reported to decrease with depth, indicating the conversion of aliphatic to aromatic carbon through decomposition (Barre et al. 2016; Upton et al. 2018). OI generally correlates with the presence of polysaccharides, consistent with the high presence of oxygen particularly in comparison to aromatic compounds. Changes in OI have therefore been proposed as a possible indicator of polysaccharide decomposition in peat (Delarue et al. 2013), although are subject to a higher degree of error than other parameters (Behar et al. 2001).

We used Rock-Eval pyrolysis to assess the heterogeneity of surface peat organic matter properties at two distances within the rooting zones of two plant species commonly found in tropical peatlands, *Campnosperma panamensis* (Standl.), a broadleaved evergreen tree, and *Raphia taedigera* (Mart.), a canopy palm. We then linked differences in organic matter properties at both distances to GHG fluxes measurements, averaged over 1 month to assess how peat chemical properties can regulate emissions. We hypothesised that: (i) carbon emissions differ with distance from the stems because of chemical and physical conditions; (ii) peat mineralization results in preferential decay of labile organic compounds; (iii) consequently the balance of production/degradation over time drives the differences in peat composition with the distance from stems; (iv) differences in organic matter inputs between plant species will result in distinct peat properties and carbon emissions.

## Methods

### Study sites

This study was conducted in a 80 km^2^ ombrotrophic peatland at Changuinola, part of the San San Pond Sak freshwater and marine wetland located in Bocas del Toro province, Panama. The peatland was initiated 4000–5000 years ago and is 8 m deep at the centre of the peat dome (Phillips et al. 1997). The peatland supports seven plant communities along a successional gradient from the coast to the central peat dome. The coastal side is dominated by *Rhizophora mangle* mangrove swamp, succeeded by *R. taedigera* dominated palm swamp, a mixed species forest swamp, *C. panamensis* forest swamp, a stunted forest swamp and a *Myrica*-*Cyrilla* bog-plain (Phillips et al. 1997). The peatland is characterised by a decline in nutrient availability towards the centre of the peat dome (Sjögersten et al. 2011).

Mean annual temperature was 26 °C in the 13 years prior to sampling, with a mean annual rainfall of 3207 mm. During the sampling period in February 2015, peat and mean air were 25 °C and rainfall was 280 mm, which are broadly representative of climatic conditions throughout the year (Wright et al. 2013b). The water table height varied during sampling but remained consistently just above the peat surface in hollows where sampling occurred, although hummocks were often raised above the water table.

### Peat and pore water sampling

**Fig. 1:**
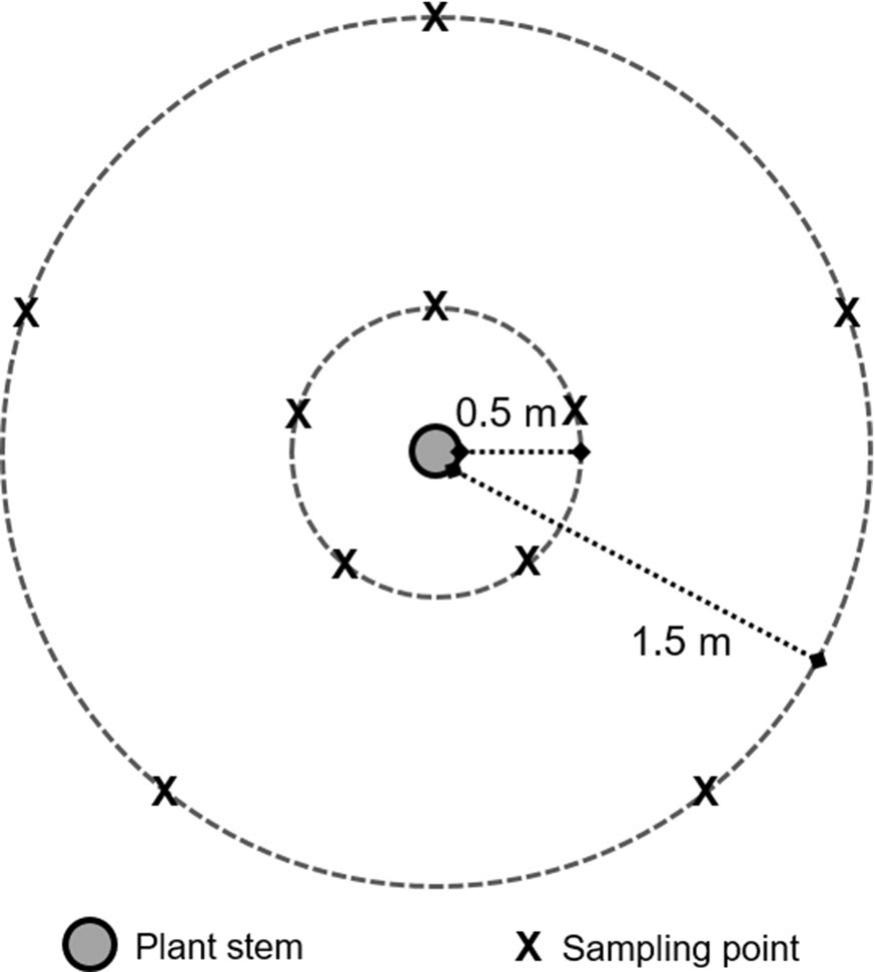
Peat sampling strategy at five points around each individual plant stem, replicated across six *C. panamensis* and six *R. taedigera* plants

Peat samples were collected from a mixed species stand of *C. panamensis* and *R. taedigera* located 600 m from the coast (09°18′13.00″N, 82°21′13.80″W). Samples were collected from under six *C. panamensis* trees and six *R. taedigera* palms selected to maximise distance to the nearest neighbouring plant in an area measuring approximately 1 ha. Peat samples were collected at two distances from plant stems, 0.5 m and at 1.5 m, to assess variation in biogeochemical properties within the rooting zone of each individual plant, particularly the effects of reduced root biomass (Fig. 1). This assumed that fine root biomass decreased with increasing distance from plant stems (Taskinen et al. 2003; Bauhus and Bartsch 1996; Mulia and Dupraz 2006), which has previously been reported in oil palm plantations (Matysek et al. 2017). Samples were not collected at greater distances due to the increased likelihood of influences from other plants including understory vegetation although this was limited, particularly under *R. taedigera* (Wright et al. 2013b). Tropical forested peatlands have previously been characterised as featuring distinct hummocks raised above the water table which are generally raised above the water table throughout the year, and hollows which are periodically inundated depending on changes in the water table. Hummocks have previously been proposed as more significant CO_2_ sources than hollows, with hollows more important CH_4_ sources during inundation (Jauhiainen et al. 2005; Comeau et al. 2013) although this is not a consistent finding (Hergoualac’h et al. 2017). The microtopography under both *R. taedigera* and *C. panamensis* featured a mix of shallow water pools and raised areas primarily driven by increased the presence of pneumatophores and roots. Sampling of both peat and GHGs was conducted in hollows at both distances to ensure that sampling was conducted in areas continually inundated.

Five samples were collected under each plant to a depth of 25 cm using a hand trowel, and combined to create a composite sample. Tree and palm height and diameter-at-breast-height (DBH) were measured for each selected plant, with DBH measured for *R. taedigera* at the highest point before the stem divided into individual palm fronds. A single peat core was collected at both distances under each plant and to a depth of 25 cm to measure fine root density. Cores were weighed and live, fine roots were (< 2 mm) separated by hand based on colour, washed and oven dried at 65 °C for 3 days. In the absence of any nearby vegetation, it was assumed that live roots were derived solely from the plant at the focal point of sampling. Dry root density was calculated using dry root weight divided by the volume of the core.

### In situ gas flux measurements

In situ gas fluxes were measured adjacent to each peat sampling site on five occasions over 1 month from hollows at each site. CH_4_ fluxes across nearby sites have previously been shown to be fairly consistent throughout the year between January and June, although CO_2_ fluxes are more variable (Wright et al. 2013b). Chambers were placed in hollows, avoiding understory vegetation which may represent an additional organic matter source. Fluxes are therefore a measure of soil respiration rather than ecosystem respiration. Gases were collected from headspaces (0.19 dm^3^) fitted with a Suba seal^®^ rubber septa (Fisherbrand, Loughborough, UK) between 10 a.m. and 4 p.m. on each sampling day, with samples collected 0, 4 and 8 min following chamber installation (Sjögersten et al. 2011). Headspace gases were mixed using a 20 ml syringe and needle and injected into pre-evacuated 12 ml glass exetainers sealed with a screw cap septum, resulting in a slight over-pressurisation (LABCO, UK). CO_2_ and CH_4_ concentrations were measured using a single injection system with a 1 ml sample loop using N_2_ as a carrier gas through a non-polar methyl silicone capillary column (GC-2014; Shimadzu, Milton Keynes, UK). A thermal conductivity detector and flame ionization detector were used to measure CO_2_ and CH_4_ respectively. Fluxes were calculated using the ideal gas law and assuming the linear accumulation of gases over time (Hogg et al. 1992). Samples that were under-pressurised at the time of analysis were discarded.

### Peat and pore water characterization

Peat sub-samples were used to characterize physiochemical properties to 25 cm depth, at 0.5 and 1.5 m from plant stems. Gravimetric moisture content was determined from the mass lost from 10 g of wet peat, oven dried at 105 °C for 24 h. Peat organic matter content was determined as the mass lost after ignition for 7 h at 550 °C in a muffle furnace. Bulk density was measured by collecting intact 10 cm × 10 cm × 25 cm sections from the peat surface, oven drying at 105 °C for 24 h, and weighing. Total peat carbon (C) and total nitrogen (N) content were determined from 0.2 g of dry, homogenised peat combusted using a total element analyser (Flash EA 1112, CE Instruments, Wigan, UK).

Dehydrogenase activity, used as a general indicator of microbial activity, was determined by the reduction of 2,3,5-triphenyltetrazolium chloride (TPC) (Ohlinger 1995). Two replicate peat samples (1.5 g dry weight) were incubated with 5 ml of 3% TPC at 30 °C for 24 h, producing triphenyl formazan (TPF). TPF was extracted by shaking with 25 ml of methanol for 2 h in the dark. The solution was then filtered in a semi-dark room and the intensity of TPF was measured at 485 nm against a range of known standards. Dehydrogenase activity was expressed in µg TPF g^−1^ h^−1^.

Peat pore water samples were collected from 25 cm depth using 10 cm long Rhizon samplers made from hydrophilic porous polymer with a pore diameter of 0.1 µm to exclude peat particles (Rhizosphere Research Products, Wageningen, The Netherlands). Samples were frozen during storage and transport. Fulvic acid to humic acid ratios in pore water, indicative of the extent of peat humification and aromaticity, were assessed by spectrophotometric analysis (Grayson and Holden 2012). Pore water samples were passed through a cellulose filter (Whatman Grade 1, 11 μm) and analysed at 465 and 665 nm (U-2010, Hitachi UV–Vis Spectrophotometer) to calculate the E_465_:E_665_ index which reflects the ratio of humic to fulvic acids and thus broadly indicates the extent of humification (Uyguner and Bekbolet 2005). Dissolved organic carbon (DOC) and total dissolved nitrogen (TDN) were measured using a TOC-V/TN analyser (Shimadzu Corp, Kyoto, Japan).

### Rock-Eval 6 pyrolysis

Peat sub-samples were analysed using a Rock-Eval 6 analyser. Freeze-dried powdered peat samples (60 mg) were heated at 300 °C for 3 min before an increase in temperature to 650 °C at a rate of 25 °C per minute in an inert N_2_ atmosphere. Residual carbon was subsequently oxidized from 300 to 850 °C at a rate of 20 °C per minute. The release of hydrocarbons during the two-stage pyrolysis process was detected by a flame ionisation detector, with an infrared cell detecting the release of CO and CO_2_ during the thermal cracking of the organic matter. Rock-Eval analysis generated a range of standard parameters including S1 (a measure of free hydrocarbons released on heating to 300 °C), S2 (hydrocarbons released on the thermal cracking of organic matter for temperatures up to 650 °C), S3CO and S3CO_2_ (the CO and CO_2_ yielded from the breakdown of kerogen), Tpk2 (the temperature associated with the highest yield of bound hydrocarbons) and total organic carbon (TOC_RE_). The Hydrogen Index (HI, mg HC g^−1^ TOC_RE6_), a measure of hydrocarbons released relative to TOC was calculated as S2 × 100/TOC_RE_. The Oxygen Index (OI, mg O_2_ g^−1^ TOC_RE6_), corresponding to the amount of oxygen released as CO_2_ relative to TOC_RE_ was calculated as S3 × 100/TOC_RE_.

Additional parameters were derived from the deconvolution of S2 pyrograms using Fityk v0.9.7, a curve fitting and data analysis program. S2 pyrograms were deconvoluted into six Gaussian signals (F1–F6) based on maximising R^2^ coefficient values. F1–F6 values have previously been attributed to organic compounds of increasing thermal stability (Disnar et al. 2003; Sebag et al. 2006). F1 and F2 correspond to thermally labile fresh plant material including simple polysaccharides, and lignin and cellulose derived compounds, respectively. F3 and F4 relate to humic compounds. F5 and F6 correspond to highly mature and recalcitrant organic matter, or charcoal (Sebag et al. 2016). Saenger et al. (2013) proposed combining these signals into three pools representing different phases of carbon thermal stability, with C_l_ representing highly labile hydrocarbon compounds (F1 and F2), C_i_ corresponding to the more stabilised carbon pool (F3), and the highly recalcitrant pool, C_p_ (F4–F6). The amount of carbon in each pool was subsequently calculated for the upper 20 cm of the peat, expressed in g C m^−2^, using TOC_RE6_ and bulk density.

A second set of indices, I and R, describing the preservation of thermally labile and highly thermostable organic matter respectively, were calculated using the approach proposed by Sebag et al. (2016) using the integrated areas under the S2 curve between given temperature nodes. Areas were calculated as 200–340 °C (A1, labile biopolymers), 340–400 °C (A2, resistant biopolymers), 400–460 °C (A3, immature geopolymers), and > 460 °C (A4, mature geopolymers). The I-index was calculated as log((A1 + A2)/A3) and the R-index was calculated as (A3 + A4)/100 and represents the contribution of mature organic matter to the S2 signal (Sebag et al. 2016).

### Statistical analysis

Statistical analyses were conducted in GenStat v17.1. Differences in CO_2_ and CH_4_ fluxes, peat properties, including Rock-Eval parameters and indices, were assessed using a linear mixed effects model fitted using residual maximum likelihood (REML) to account for variable dependence between sampling plots. CO_2_ and CH_4_ fluxes, dehydrogenase activity, and the proportional pools C_l_, C_i_ and C_p_ were log_10_-transformed to meet assumptions of normality, tested using Shapiro–Wilk tests. Significance was assessed at p ≤ 0.05. Relationships between key environmental variables and mean GHG fluxes (averaged over the sampling period) were assessed using Principal Component Analysis (PCA), based on correlation matrices. Backwards stepwise elimination regression was then used to identify significant relationships between Rock-Eval parameters and indices, and logged CO_2_ and CH_4_ fluxes. The maximal model included C_l_, C_i_ and C_p_ pool carbon stocks, HI, OI, S2, S3CO, S3CO_2,_ and TpkS2. Full ANOVA tables are reported in supplementary materials.

## Results

### Vegetation, peat and pore water properties

*Campnosperma panamensis* trees had a mean height of 15.0 m, and DBH of 41.6 cm, with fine root density ranging from 8.2 to 10.6 mg cm^−3^ across the rooting zone (Table 1). Peats were characterised by low bulk density (0.08–0.14 g cm^−3^) and consistently high moisture (89.0%). Peats were acidic, and weakly reducing (306–311 mV), with low electrical conductivity (115–124 µS). Microbial activity in the peats was broadly comparable to previous measurements ex situ (3.1 µg TPF g^−1^ h^−1^) (Girkin et al. 2018a). Organic matter content and total carbon was high, but low nitrogen resulted in C:N ratios of 16.8–17.7. *C. panamensis* pore water had maximum DOC and TDN content at 0.5 m (166.8 and 22.1 mg l^−1^). E_465_:E_665_, indicative of the condensation of aromatic carbon, increased slightly at 1.5 m (9.9) compared to 0.5 m (8.5).

**Table 1:** Tree characteristics, peat and pore water properties of *C. panamensis* and *R. taedigera*

Species	*C. panamensis*		*R. taedigera*	
Distance (m)	0.5	1.5	0.5	1.5
Height (m)	15 ± 0.8	–	8.3 ± 3.4	–
DBH (cm)	41.6 ± 2.6	–	30.2 ± 3.7	–
Fine root density (mg cm^−3^)	10.6 ± 2.2	8.2 ± 1.2	8.1 ± 1.5	6.5 ± 1.5
Bulk density (g cm^−3^)	0.08 ± 0.01	0.14 ± 0.05	0.10 ± 0.01	0.08 ± 0.01
Gravimetric moisture content (%)	89.0 ± 1.0	89.0 ± 0.5	89.2 ± 0.9	90.5 ± 0.5
pH	3.95 ± 0.05	3.88 ± 0.14	4.16 ± 0.06	3.94 ± 0.10
Conductivity (µS)	112 ± 25	124 ± 29	70 ± 12	75 ± 10
Redox potential (mV)	311 ± 9	306 ± 5	304 ± 4	306 ± 5
Dehydrogenase (µg TPF g^−1^ h^−1^)	3.1 ± 0.2	3.1 ± 0.3	3.6 ± 0.3	2.9 ± 0.3
Total carbon (%)	46.0 ± 1.5	45.7 ± 3.4	43.6 ± 1.7	41.0 ± 4.0
Total nitrogen (%)	2.8 ± 0.1	2.6 ± 0.3	2.6 ± 0.2	2.5 ± 0.3
C:N	16.8 ± 0.7	17.7 ± 0.9	16.8 ± 0.4	16.2 ± 0.4
Organic matter content (%)	94.7 ± 0.6	96.3 ± 0.7	88.7 ± 2.6	92.6 ± 2.3
DOC (mg l^−1^)	166.8 ± 19.1	149.8 ± 17.2	184 ± 28.6	188.2 ± 31.2
TDN (mg l^−1^)	22.1 ± 3.1	21.1 ± 1.9	21 ± 3.3	22.1 ± 3.6
E_465_:E_665_	8.5 ± 0.4	9.9 ± 0.7	8.7 ± 1.1	8.9 ± 0.7

Means ± 1 SEM (n = 6)

*Raphia taedigera* palms were smaller than *C. panamensis* trees with a mean height of 8.3 m and significantly lower DBH (F_1,10_ = 6.27, p = 0.03). Fine root density decreased with distance and was lower than *C. panamensis*, but these differences were not significant. *R. taedigera* peats were characterised by low bulk density (0.06–0.10 g cm^-3^) and high moisture content. Peats were acidic, with pH declining from 4.16 at 0.5 m to 3.94 at 1.5, and had low conductivity (70–75 µS) and a weakly reducing redox potential. At 0.5 m from the stem, mean dehydrogenase activity was higher than under *C. panamensis* peats (3.6 µg TPF g^−1^ h^−1^) but decreased to 2.9 µg TPF g^−1^ h^−1^ at 1.5 m. Organic matter content was lower at 0.5 m (88.7%) compared to 1.5 m (92.6%), matching the trend observed under *C. panamensis* but total carbon was broadly similar at both distances (43.6% and 41%). Total nitrogen and C:N both declined at 1.5 m. Pore water collected from under *R. taedigera* at both distances was characterised by high DOC but low TDN content, and consistent E_465_:E_665_ ratios which indicated humified organic matter.

Of these changes, pH declined significantly under both species at 1.5 m from plant stems (F_1,10_ = 5.16, p = 0.049). Additionally, organic matter content was significantly higher at 1.5 m compared to 0.5 m (F_1,10_ = 10.80, p = 0.01).

### In situ CO_2_ and CH_4_ fluxes

**Fig. 2:**
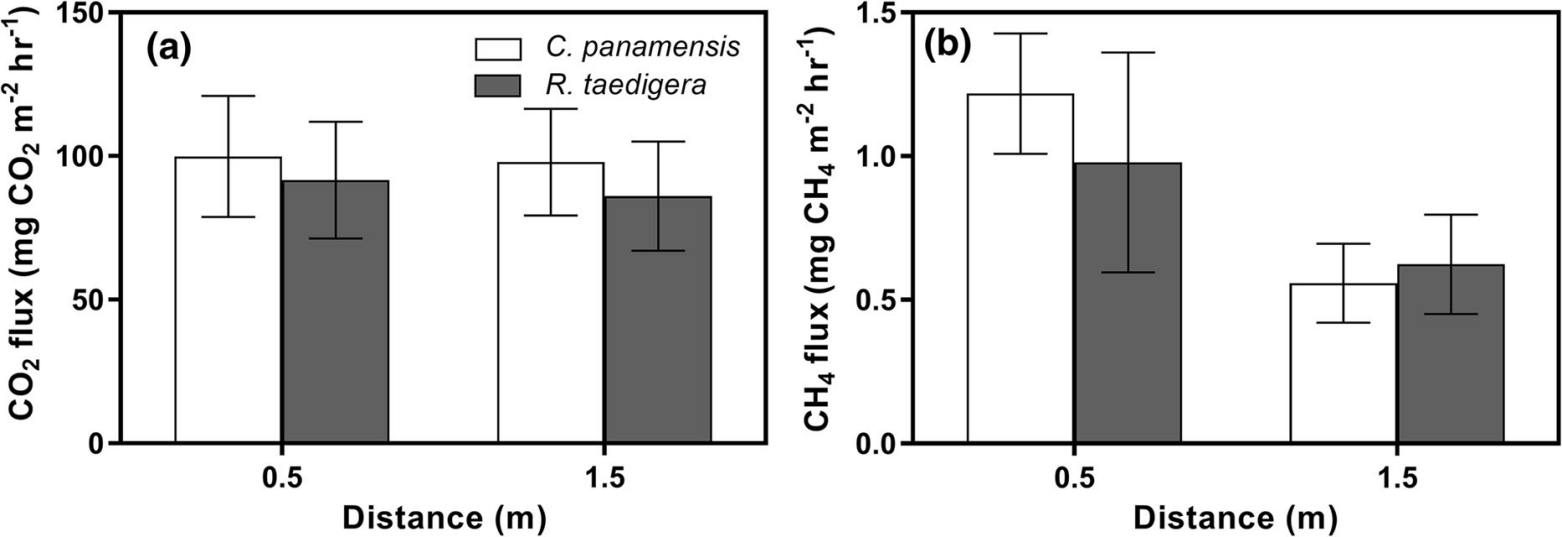
**a** CO_2_, and **b** CH_4_ fluxes at 0.5 m and 1.5 m from *C. panamensis* and *R. taedigera* plant stems. Means ± 1 SEM (n = 6)

Mean CO_2_ fluxes were consistently lower at 0.5 m under *C. panamensis* (84.50 mg CO_2_ m^−2^ h^−1^), increasing to 108.22 mg CO_2_ m^−2^ h^−1^ at 1.5 m (Fig. 2a). CO_2_ fluxes at 0.5 m were higher under *R. taedigera* (124.50 mg CO_2_ m^−2^ h^−1^), decreasing to 119.92 mg CO_2_ m^−2^ h^−1^ at 1.5 m, although these differences were not significant (F_1,37.6_ = 0.02, p = 0.88). CO_2_ fluxes varied significantly during the sampling period, ranging from 291.98 to 32.52 mg CO_2_ m^−2^ h^−1^ (F_4,33.8_ = 7.32, p < 0.001).

In contrast, CH_4_ fluxes were significantly higher at 0.5 m compared to 1.5 m under both species (F_1,35_ = 6.03, p = 0.02). Mean fluxes under *C. panamensis* decreased from 1.25 to 0.56 mg CH_4_ m^−2^ h^−1^, and decreased under *R. taedigera* from 1.00 to 0.75 mg CH_4_ m^−2^ h^−1^ but the difference between species was not significant (F_1,9.9_ = 0.06, p = 0.82). CH_4_ fluxes also varied significantly over the course of the sampling period (F_4,29.5_ = 3.92, p = 0.01).

### Bulk organic matter properties

S1, indicative of low weight organic compounds, was low but has previously been suggested as being of minimal importance in soils (Table 2) (Disnar et al. 2003). S2 under *C. panamensis* decreased slightly from 123 to 118 mg g^–1^ at 1.5 m, whereas S3CO and S3CO_2_ increased slightly. TpkS2, matched the trend in S2 values, decreasing at 1.5 m. The lower TpkS2 at 1.5 m (417 °C) is typical for the thermal breakdown of humic substances (Disnar et al. 2003). TOC was high (40–41%) but varied little within the rooting zone. HI decreased from 310 to 290 mg HC g^−1^ TOC_RE6_ at 1.5 m, with a concomitant increase in OI from 200 to 221 mg O_2_ g^−1^ TOC_RE6_, although these differences were not significant.

**Table 2:** Selected Rock-Eval 6 parameters

Species	*C. panamensis*		*R. taedigera*	
Distance (m)	0.5	1.5	0.5	1.5
S1 (mg g^−1^)	33 ± 3	31 ± 2	31 ± 2	35 ± 2
S2 (mg g^−1^)	123 ± 9	118 ± 6	113 ± 8	125 ± 2
S3CO (mg g^−1^)	19 ± 2	22 ± 1	18 ± 1	17 ± 1
S3CO_2_ (mg g^−1^)	78 ± 6	89 ± 2	701 ± 5	73 ± 5
TpkS2 (°C)	426 ± 2	417 ± 9	414 ± 9	422 ± 2
TOC_RE_ (%)	39 ± 3	41 ± 1	37 ± 2	40 ± 2
HI (mg HC g^−1^ TOC_RE6_)	310 ± 5	290 ± 14	306 ± 13	316 ± 14
OI (mg O_2_ g^−1^ TOC_RE6_)	200 ± 9	221 ± 11	191 ± 5	185 ± 10

Means ± 1 SEM (n = 6)

*R. taedigera* peats showed significant differences in several Rock-Eval parameters compared to *C. panamensis*. In contrast to *C. panamensis*, S2 increased to 125 mg g^−1^ at 1.5 m from 113 mg g^−1^ at 0.5 m. There was a significant difference in S3CO (F_1,10_ = 4.97, p = 0.049) and S3CO_2_ (F_1,10_ = 5.16, p = 0.049) between species, with both higher under *C. panamensis*. Under *R. taedigera*, TpkS2 increased from 414 °C at 0.5 m to 422 °C at 1.5 m, showing the opposite trend to *C. panamensis*. TOC_RE6_ was also somewhat lower, ranging from 37 to 40% but this difference was not significant. In contrast to *C. panamensis* peats, HI increased from 306 mg HC g^−1^ TOC_RE6_ at 0.5 m to 316 mg HC g^−1^ TOC_RE6_ at 1.5 m, while OI decreased significantly from 191 mg O_2_ g^−1^ TOC_RE6_ at 0.5 m to 185 mg O_2_ g^−1^ TOC_RE6_ at 1.5 m (F_1,10_ = 6.37, p = 0.03).

### Organic matter indices and carbon stocks

**Fig. 3:**
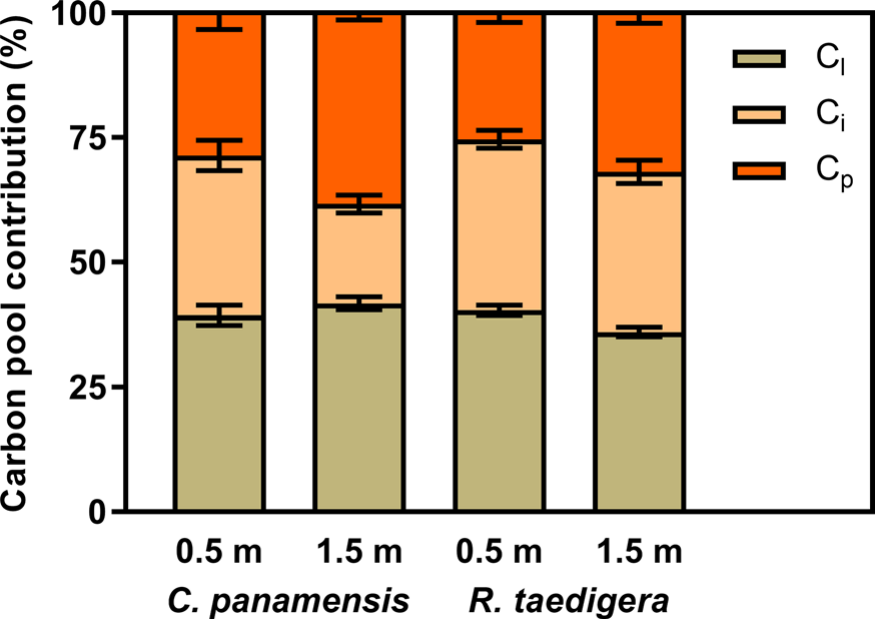
Proportions of peat labile (C_l_), intermediate (C_i_) and passive (C_p_) carbon pools for *C. panamensis* and *R. taedigera* in peats 0.5 m and 1.5 m from plant stems. Means ± 1 SEM (n = 6)

**Fig. 4:**
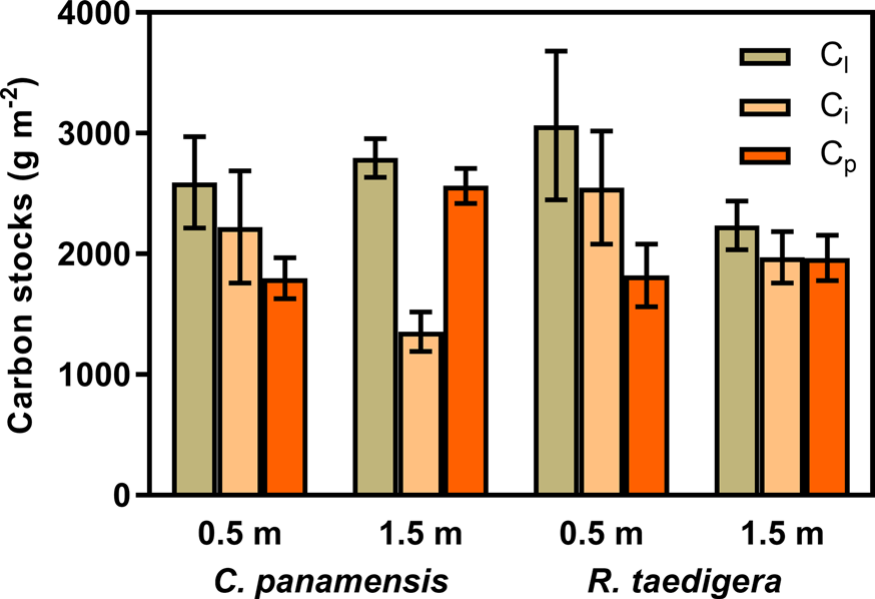
Carbon stocks associated with labile (C_l_), intermediate (C_i_) and passive (C_p_) pools for *C. panamensis* and *R. taedigera* in peats 0.5 m and 1.5 m from plant stems. Means ± 1 SEM (n = 6)

Under both *C. panamensis* and *R. taedigera* and at both distances, the peat labile carbon pool (C_l_) was consistently the largest (Fig. 3), accounting for 36–42% of hydrocarbons released during pyrolysis (S2). Labile carbon stocks ranged from 2236 to 3065 g m^−2^ (Fig. 4). Moreover, the pool exhibited contrasting trends between species. For *C. panamensis*, C_l_ was greater a 1.5 m compared to 0.5 m, with the opposite trend observed for *R. taedigera*. The intermediate carbon pool (C_i_) ranged from 20 to 34%, the equivalent of 1354–2549 g m^−2^ carbon stocks. These stocks were significantly higher at 0.5 m compared to 1.5 m (F_1,10_ = 1.18, p = 0.02). Carbon stocks associated with the passive pool (C_p_) also varied significantly with distance from plant stems, increasing at 1.5 m compared to 0.5 m (F_1,10_ = 6.85, p = 0.03), accounting for 25–35%.

**Fig. 5:**
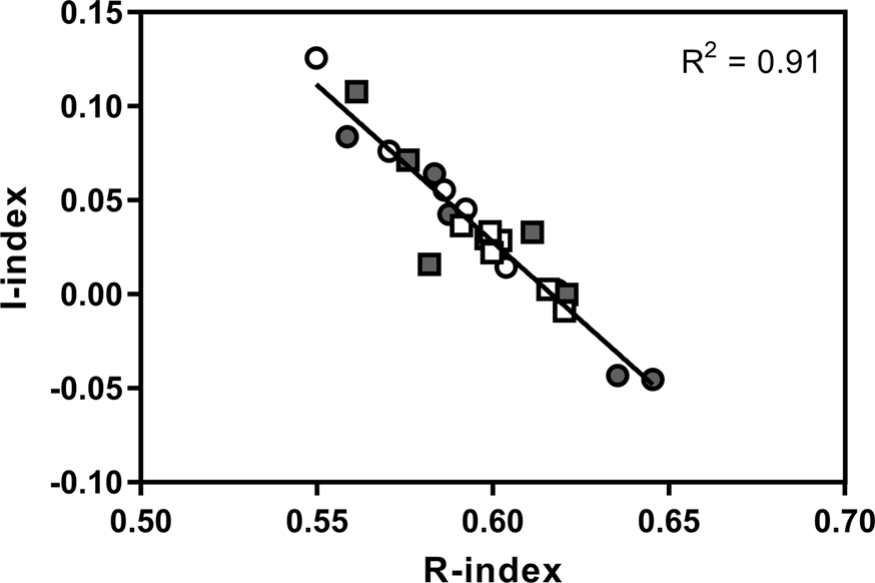
I and R indices for *C. panamensis* (white) and *R. taedigera* (grey) peats at 0.5 m (filled square) and 1.5 m (filled circle) from plant stems

The I-index, describing thermally labile organic matter, ranged from 0.017 to 0.057, and the R-index, describing highly thermostable organic matter, ranged from 0.058 to 0.060 (Fig. 5). *C. panamensis* peats at 0.5 m have low I and high R indices, as were *R. taedigera* peats at 1.5 m. In contrast, *C. panamensis* peats at 1.5 m are characterised by high I and low R indices. However, differences for both I and R indices between species, and with distance, were not found to be significant.

### Organic matter properties and CO_2_ and CH_4_ fluxes

**Fig. 6:**
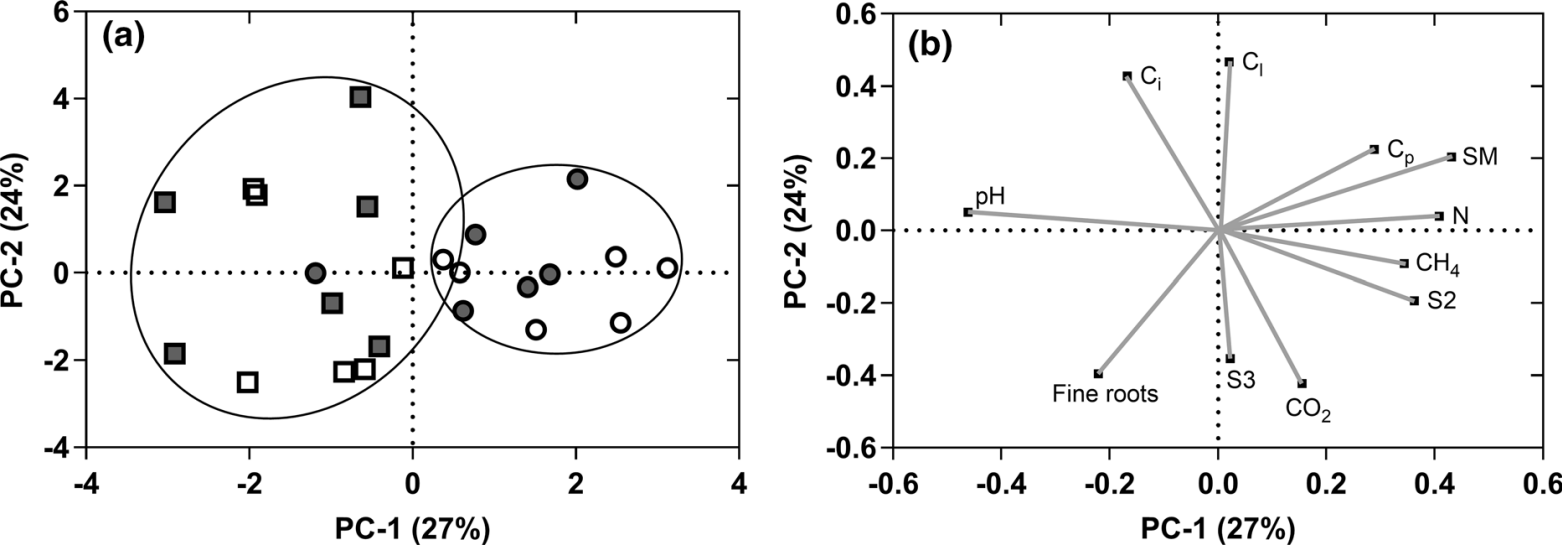
**a** Scores for *C. panamensis* (white) and *R. taedigera* (grey) peats at 0.5 m (filled square) and 1.5 m (filled circle) from plant stems. **b** Loadings from PCA for all species and peats. Combined PC1 and PC2 account for 51% of variance

**Fig. 7:**
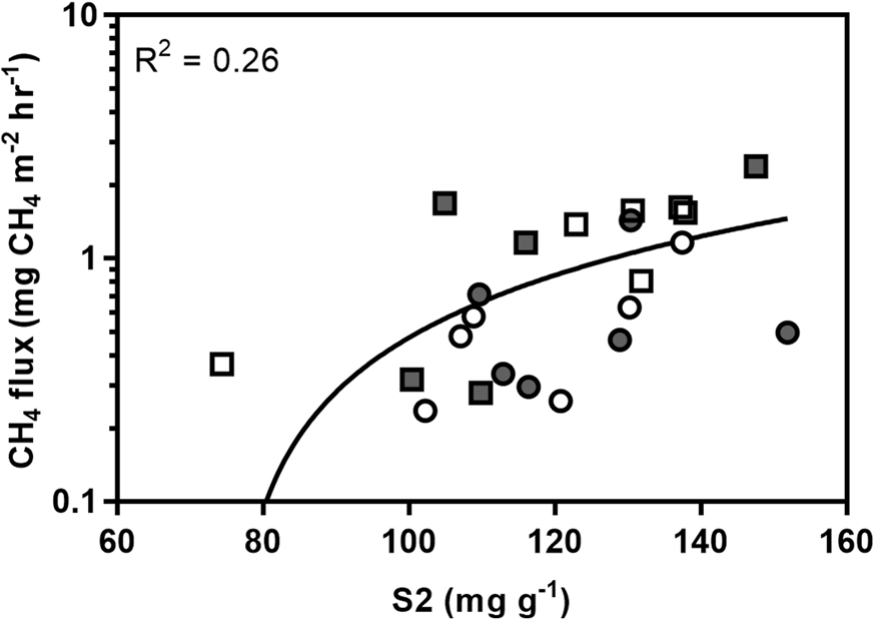
Linear regression of S2 and logged mean CH_4_ fluxes for *C. panamensis* (white) and *R. taedigera* (grey) peats at 0.5 m (filled square) and 1.5 m (filled circle) from plant stems

The scores and loadings of the first and second principal components accounted for the majority of the variance (51%) (Fig. 6). Separation along the first principal component is mainly driven by pH, total nitrogen (N), moisture content (SM) and S2, and along the second component by C_l_, and fine root biomass. Peats are predominantly clustered by distance from plant stems, with peats from 1.5 m having reduced variation compared to peat from 0.5 m. Linear regression identified a significant relationship between S2 and mean CH_4_ efflux (F_1,22_ = 9.25, p = 0.006, R^2^ = 26.4, Fig. 7), indicating that overall substrate availability, rather than any specific carbon pool, was important in driving fluxes. No significant variables were identified for driving CO_2_ efflux.

## Discussion

### Rooting zone trends in peat biogeochemistry and greenhouse gas fluxes

Changes in fine root biomass with increased distance from plant stems were relatively small and not significant. This may reflect sampling in hollows around plant stems which may feature reduced root biomass compared to hummocks which are formed from roots and fallen leaf litter (Jauhiainen et al. 2005).

pH and organic matter both declined with distance from plant stems under both species, although these differences were only significant for *R. taedigera*. In general, differences in peat properties between species were reduced compared to previous studies, with only pH differing significantly at 0.5 m. This lack of significant differences is most likely because this study was conducted in a mixed forest rather than monodominant stand which means that peat properties are likely to be influenced to a lesser extent by the previously reported gradient in nutrient availability and microbial communities (Sjögersten et al. 2011; Cheesman et al. 2012; Troxler et al. 2012). Additionally, although individual plants were selected to minimise the presence of nearby understory and canopy vegetation, their influence on peat properties is likely to be limited but cannot be fully discounted.

Aerobic decomposition of organic matter under low water table conditions may drive increases in pH through decarboxylation of organic anions, which involves protonation (Yan et al. 1996). Lower pH with increasing distance from the stems may therefore be associated with reduced decomposition rates, which is supported by increased organic matter content and C_i_ carbon stocks. Previous studies have indicated that carbon inputs within the rooting zone can substantially increase decomposition, with species specific effects (Kuzyakov and Cheng 2001; Cheng et al. 2003). Changes in pH have been identified as significant drivers of changes to soil microbial communities (Shi et al. 2011). The lack of significant difference in dehydrogenase activity, used as an overall measure of microbial activity, may be associated with the high organic matter content, as activity is unlikely to be carbon substrate limited.

CO_2_ fluxes did not decline with distance under either species, possibly due to limited differences in root density. Declines in root biomass might have been expected to be associated with lower root-derived CO_2_. Estimates of root respiration in tropical sites are variable, accounting for up to two-thirds of total CO_2_ flux from forested peatlands in Malaysia and Panama (Melling et al. 2013; Girkin et al. 2018c), but varying from 24 to 61% in Amazon forests (Silver et al. 2005; Metcalfe et al. 2007). CO_2_ emissions can also be driven by microbial utilisation of root exudates and peat organic matter, and the oxidation of CH_4_ (Wright et al. 2013a).

In contrast to CO_2_ fluxes, CH_4_ varied significantly within the rooting zone under both species, being significantly higher at 0.5 m compared to 1.5 m. Previous work in rice has suggested that labile root exudates are the most substrate for methanogenic *Archaea* (Lu and Conrad 2005) and inputs may be greater close to plant stems. Methanogen abundance has previously been found to be higher in organic matter rhizosphere in a wetland soil (Pajares et al. 2016) and, in conjunction with variation in OM properties, may account for the observed fluxes. The smaller differences in CH_4_ fluxes between *R. taedigera* may in part be driven by greater inputs of root oxygen which drive the oxidation of CH_4_ (Hoyos-Santillan et al. 2016a).

### Organic matter properties and thermal stability

Deconvolution of the S2 pyrograms into F1–F6 fractions has previously been used to assess the thermal stability of organic matter through changes in the relative proportions of pools of organic polymers and carbon stocks (Saenger et al. 2013). However, there are significant drawbacks in assessing proportional changes in pool sizes due to a decrease in one pool increasing the size of one or both of the other pools. Previous comparisons of F1–F6 with FTIR (Delarue et al. 2013; Biester et al. 2014) and ^13^C NMR (Albrecht et al. 2015), indicate only a partial correlation with distinct functional groups as different biomolecules, such as lignin and cellulose, frequently pyrolyse at the same temperature (Disnar et al. 2003). Changes in C_l_, C_i_ and C_p_ therefore indicate broad scale patterns in thermal stability driven by organic matter degradation rather than changes in any one specific functional group (Sebag et al. 2016). Surface peats at all distances showed large overall carbon stocks with a significant contribution to the overall pool size by labile biopolymers. The labile pool is highly sensitive to mineralisation and may therefore be particularly vulnerable to loss due to climate change. CH_4_ fluxes have been shown to be highly impacted by increased temperature at the site, which can deplete labile carbon, with the precise effect differing between peats derived from contrasting botanical origins (Sjögersten et al. 2018). These results also demonstrate that carbon stocks can be highly heterogenous on relatively short spatial scales, in addition to previously reported variation between different plant communities (Upton et al. 2018).

High rates of microbial activity may have been expected to rapidly deplete C_l_, but it is possible that root exudates and recent root necromass represent a significant and regular carbon input under both species thereby maintaining a large overall pool. C_i_ and C_p_ combined indicate a large pool of humified compounds, most likely derived gradual decomposition of recent litter inputs (Disnar et al. 2003). Overall, deconvolution indicates that rooting zone peats are highly heterogeneous but generally feature a large labile carbon pool, likely derived from fresh litter inputs, in addition to quantities of thermostable biopolymers and humic substances derived from ongoing decomposition processes.

There was no consistent significant pattern in I and R indices with distance from plant stems or peat botanical origin. This may be because this approach for assessing organic matter dynamics is better suited to indicting broader temporal or spatial changes with depth (Sebag et al. 2016), with comparatively smaller changes occurring over short distances in a highly heterogeneous landscape. For example, these indices have previously been applied in demonstrating variation along a beech plantation chronosequence and changes during composting. The decrease in the contribution of biopolymers (A1 and A2) occurs simultaneously with increases in geopolymers (A3 and A4) resulting in a high correlation between the indices (R^2^ = 0.91), demonstrating the relationship between decreasing relative contributions from labile organic matter, and increases in recalcitrant pools (Sebag et al. 2016).

Previous studies have demonstrated that OI and HI are reliable indicators of soil organic matter maturation, although changes are partially relative (Disnar et al. 2003; Hetenyi et al. 2005; Saenger et al. 2013), with high HI values (over 300 mg HC g^−1^ TOC) driven by minimally degraded plant tissues rich in polysaccharides, and lower values associated with woody materials with high cellulose and lignin (Marchand et al. 2008). During humification, HI decreases due to increasing aromaticity and dehydrogenation, and OI increases through oxidation, although high values can also be associated with polysaccharides which are relatively rich in oxygen (Hetenyi et al. 2006; Carrie et al. 2012; Sebag et al. 2016). This has been found to be particularly apparent in subsurface peat layers where TpkS2 increases and HI decreases due to the reduced contribution of fresh litter inputs with high carbohydrate, protein and lipid contents (Disnar et al. 2003; Upton et al. 2018). Alternatively, reduced HI may be caused by an increased proportion of highly aromatic organic matter, which also has low HI values (Saenger et al. 2013).

### Small scale variation in organic matter properties

Contrasts in peat organic matter properties with distance from stems between species are likely to reflect the interaction of a range of biotic and abiotic environmental variables. Leaf, stem and shoot litter from *C. panamensis* and *R. taedigera* litter have previously explained differences in litter decomposition rates within depth profiles (Hoyos-Santillan et al. 2016b). In surface peats *R. taedigera* litter decomposes faster than *C. panamensis* litter, possibly because of higher nitrogen content. However, in waterlogged soils the inhibition of aerobic ligninolytic microorganisms reduces decomposition of lignin rich *R. taedigera* litter (Zeikus 1981). Differences in plant morphology may also account for some variation in observed organic matter properties. *C. panamensis* has large buttress roots compared to large surface root mats and pneumatophores under *R. taedigera*. *C. panamensis* has small leaves which are likely to represent a more regular carbon input, compared to large palm fronds from *R. taedigera* which will represent a single larger carbon pulse in surface peats (Wright et al. 2013b). Differences in rooting structure can, in turn, strongly influence peat surface microtopography resulting in distinctive raised hummocks and slight depressions (hollows). While the former are often above the water table, hollows can undergo distinct phases of immersion and exposure depending on precipitation (Jauhiainen et al. 2005). Previously the water table at the site has been reported to change by up to 20 cm (Wright et al. 2013b). In part, the influence of microtopography on fluxes was controlled by sampling only in hollows which remained continually inundated during the sampling period with the water table remaining just above the peat surface. Differences in organic matter properties, for example relative changes in C_l_, C_i_ and C_p_, between peats derived from contrasting botanical origins may indeed be more pronounced between hummocks and hollows in part due to contrasts in litter decomposition under fluctuating aerobic vs anoxic conditions (Jauhiainen et al. 2005).

Vegetation is known to strongly determine microbial communities with both plant species and developmental stage and health known to be important biotic drivers (Berg and Smalla 2009). Work in temperate and boreal peatlands has indicated that differences in botanical composition are associated with differences in bacterial communities, with resultant effects on CH_4_ cycling (Borga et al. 1994; Robroek et al. 2015), while work in tropical forests has similarly indicated that microbial communities can vary along plant diversity gradients with C:N (Carney and Matson 2006) and phosphorous availability (Troxler et al. 2012) identified as a key driver of changes in community composition.

In this study the observed contrasting trends in peat organic matter properties with distance under each species are therefore likely to be driven by previously identified contrasts in litter properties, plant morphologies, microtopography, peat biogeochemical properties (particularly pH and C:N and the relative size of carbon pools), and microbial communities. The greater variability in peat properties at 0.5 m compared to 1.5 m likely reflects the role of changes in fine root biomass, which can mediate peat pH and other properties (including redox potential and conductivity) through species specific inputs of exudates (Girkin et al. 2018a, b) and oxygen (Hoyos-Santillan et al. 2016a).

### Linking organic matter properties and greenhouse gas fluxes

CH_4_ fluxes were not linked to any individual carbon pool but rather the total size of the relatively labile pool as measured by S2. As methanogens are only able to utilise a limited number of substrates, of which acetate is the most important (Ferry 1992), other microbial groups are likely to be mediating the availability of substrates for methanogenesis. This result highlights the importance of differences in vegetation as a driver of net CH_4_ fluxes from peatlands, as changes in dominant vegetation type will alter root and litter inputs, changing substrate availability for methanogenesis.

In contrast to CH_4_ production, no significant relationships between CO_2_ fluxes and peat properties were identified. This may indicate that environmental variables not measured were most responsible for driving CO_2_ fluxes rather than substrate availability. In addition, CO_2_ fluxes may have been driven to a significant degree by either the oxidation of CH_4_ or root respiration (Kuzyakov and Larionova 2006; Girkin et al. 2018c). Generally, surface peat between 0–30 cm is the main source of CO_2_ and CH_4_ production but some is produced at significant depth and will therefore be associated with different trends in organic chemistry (Wright et al. 2011). Gaseous transport via plant aerenchyma may also reduce surface fluxes of both gases (Pangala et al. 2017). As a consequence, ex situ incubation experiments may represent a useful approach for fully elucidating the relationships between organic matter properties and the regulation of decomposition and GHG production.

## Conclusion

Peats derived from *C. panamensis* and *R. taedigera* display significant heterogeneity of biogeochemical properties (pH, total carbon, and organic matter content) within the rooting zone. Changes in the three carbon pools derived from S2 pyrograms, combined with trends in HI and OI highlight how the different litter properties of *C. panamensis* and *R. taedigera* interact with the environment to drive decomposition. Surface peats feature a large stock of readily mineralisable labile carbon (up to 3065 g m^−2^, equivalent to 42% of surface carbon stocks) which may be particularly vulnerable to climate change. Net CH_4_ but not CO_2_ fluxes were closely associated to the overall size of the organic matter pool, rather than any one specific group of biomolecules. Collectively, these results demonstrate tropical peats derived from beneath contrasting vegetation types can exhibit significant small scale variation in soil organic matter properties which can influence GHG emissions. However, further assessments under controlled conditions are required to discount the heterogeneous contributions of plant roots and environmental conditions in situ and indicate additional relationships between GHG fluxes and peat organic matter properties. Our findings lead us to propose that changes in dominant vegetation caused by climate or land use change are likely to affect CH_4_ effluxes in tropical peatlands if associated with changes in organic carbon pool size.

## Electronic supplementary material

Below is the link to the electronic supplementary material.

Supplementary material 1 (PDF 67 kb)
