# Supplementary material for: Spatial variability of organic matter properties determines methane fluxes in a tropical forested peatland
Source: Biogeochemistry. 2018 Nov 26;142(2):231–45. doi: 10.1007/s10533-018-0531-1 (PMC6383829; doi:10.1007/s10533-018-0531-1)
Supplement: Supplementary file 1 — Supplementary material 1 (PDF 67 kb) [file 10533_2018_531_MOESM1_ESM.pdf]

Supplementary materials

Table S3: Linear mixed effects models assessing peat and pore water characteristics.

	Distance				Species				Distance * Species			
	**F-statistic**	**d.f.**	**p**	**SED**	**F-statistic**	**d.f.**	**p**	**SED**	**F-statistic**	**d.f.**	**p**	**SED**
**Height**		-			3.67	1,10	0.08	3.48		-		
**DBH**		-			*6.27*	*1,10*	*0.03*	*4.57*		-		
**Fine root density**	3.35	1,10	0.10	1,13	1.05	1,10	0.33	2.03	0.12	1,10	0.73	2.08
**Bulk density**	0.31	1,10	0.59	0.03	0.67	1,10	0.43	0.03	1.94	1,10	0.19	0.04
**Soil moisture content**	1.43	1,10	0.26	0.48	0.62	1,10	0.45	0.98	2.09	1,10	0.18	0.95
**pH**	*5.16*	*1,10*	*0.049*	*0.06*	1.30	1,10	0.28	0.12	1.17	1,10	0.30	0.12
**Conductivity**	0.27	1,10	0.62	13.6	3.21	1,10	0.10	25.9	0.02	1,10	0.89	25.9
**Redox potential**	0.08	1,10	0.78	3.85	0.22	1,10	0.65	7.62	0.64	1,10	0.44	7.51
**Log dehydrogenase**	2.86	1,10	0.12	1.07	0.18	1,10	0.68	1.07	1.93	1,10	0.20	0.10
**Total carbon**	0.8	1,10	0.39	0.85	0.98	1,10	0.33	4.60	0.27	1,10	0.62	3.52
**Total nitrogen**	0.45	1,10	0.52	0.11	0.10	1,10	0.61	0.32	1.29	1,10	0.82	0.27
**C:N**	0.30	1,10	0.84	0.58	3.26	1,10	0.30	0.74	0.82	1,10	0.28	0.90
**Organic matter content**	*10.8*	*1,10*	*0.01*	*0.84*	4.16	1,10	0.07	2.40	1.91	1,10	0.20	2.09
**DOC**	0.10	1,10	0.76	20.4	0.95	1,10	0.35	28.5	0.27	1,10	0.61	32.9
**TDN**	0.00	1,10	0.98	2.08	0.00	1,10	0.99	3.74	0.27	1,10	0.61	3.84
**E_{465}:E_{665}**	1.75	1,10	0.22	0.64	0.19	1,10	0.68	0.85	0.86	1,10	0.38	1.01

Table S4: Linear mixed effects models assessing CO_2 and CH efflux over time and with distance from plant stems.

	F-statistic	d.f.	p	S.E.D
**logCO_2**				
Day	*7.32*	*4,33.8*	*<0.001*	*0.12*
Distance	0.02	1,37.6	0.88	0.06
Species	0.05	1,10.2	0.83	0.09
Distance*Day	0.24	4,38.7	0.91	0.15
Species*Day	2.21	4,34.3	0.09	0.18
Distance*Species	0.9	1,39.0	0.35	0.10
Distance*Species*Day	0.8	0.8,40.3	0.53	0.22
**logCH_4**				
Day	*3.92*	*4,29.5*	*0.01*	*0.15*
Distance	*6.03*	*1,35*	*0.02*	*0.09*
Species	0.06	1,9.9	0.82	0.16
Day*Distance	0.65	4,35.9	0.63	0.21
Day*Species	1.25	4,30.5	0.31	0.23
Distance*Species	0.73	1,36.3	0.40	0.17
Day*Distance*Species	0.89	4,37.3	0.48	0.31

Table S5: Linear mixed effects models assessing differences in Rock-Eval 6 parameters with distance from plant stems and between species.

	**Distance**				**Species**				**Distance*Species**			
	**F-statistic**	**d.f.**	**p**	**S.E.D**	**F-statistic**	**d.f.**	**p**	**S.E.D**	**F-statistic**	**d.f.**	**p**	**S.E.D**
**S1**	0.06	1,10	0.81	2.43	0.09	1,10	0.77	2.47	1.12	1,10	0.32	3.46
**S2**	0.27	1,10	0.62	7.36	0.03	1,10	0.87	7.72	1.34	1,10	0.27	10.60
**S3CO**	0.39	1,10	0.55	1.14	*4.97*	1,10	*0.049*	*1.33*	*4.92*	*1,10*	*0.049*	*1.71*
**$S3CO_2$**	2.63	1,10	0.14	4.26	*5.16*	*1,10*	*0.049*	*5.14*	0.95	1,10	0.35	6.46
**TpkS2**	0.02	1,10	0.90	5.93	0.25	1,10	0.63	6.80	2.26	1,10	0.16	8.81
**TOC_{RE}**	0.66	1,10	0.44	2.43	0.62	1,10	0.45	2.15	0.10	1,10	0.76	3.31
**HI**	0.17	1,10	0.69	12.2	0.75	1,10	0.41	12.4	1.51	1,10	0.25	17.3
**OI**	0.71	1,10	0.42	8.89	*6.37*	*1,10*	*0.03*	*8.92*	2.36	1,10	0.16	12.6
**$logC_l$**	0.53	1,10	0.49	1.02	2.35	1,10	0.16	1.05	*6.39*	*1,10*	*0.03*	1.05
**$logC_i$**	*16.52*	*1,10*	*0.002*	*1.07*	*8.42*	*1,10*	*0.02*	*1.10*	*8.60*	*1,10*	*0.02*	*1.12*
**$logC_p$**	*14.28*	*1,10*	*0.004*	*1.07*	3.92	1,10	0.08	1.07	0.32	1,10	0.59	1.12
**C_l stock**	0.89	1,10	0.37	332.0	0.01	1,10	0.92	430.4	2.42	1,10	0.15	518.9
**C_i stock**	*1.18*	*1,10*	*0.02*	*257.0*	1.18	1,10	0.30	443.5	0.32	1,10	0.59	457.1
**C_p stock**	*6.85*	*1,10*	*0.03*	*173.8*	1.78	1,10	0.21	214.9	3.20	1,10	0.10	266.2
**I index**	0.15	1,10	0.71	0.02	0.21	1,10	0.65	0.02	3.98	1,10	0.07	0.02
**R index**	0.23	1,10	0.65	0.01	0.12	1,10	0.74	0.01	4.69	1,10	0.06	0.01
